# Natural history of familial cerebral cavernous malformations: the CCM_Italia cohort study

**DOI:** 10.3389/fneur.2025.1668098

**Published:** 2026-01-05

**Authors:** Silvia Lanfranconi, Elisa Scola, Deborah Novelli, Anna Poggesi, Francesca Pescini, Marco Pavanello, Ferruccio Romano, Quintino Giorgio D’Alessandris, Walter Marani, Francesco Signorelli, Giorgio Iaconetta, Giovanni Torelli, Enrico Fainardi, Mariasavina Severino, Luigi Gianmaria Remore, Giulio Andrea Bertani, Giorgio Conte, Valeria Capra, Antonella Vasamì, Enrico Nicolis, Giorgia Contino, Dario Ronchi, Maria Chiara Palmieri, Alessandra Previtali, Pier Paolo Mattogno, Carmelo Lucio Sturiale, Maria Elena Solarino, Rita Caliulo, Maria Teresa Bozzi, Filippo Fratini, Elisa R. Zanier, Roberto Latini, Jennifer Marie Theresia Anna Meessen, Marco Locatelli, and the CCM_Italia investigators

**Affiliations:** 1 Department of Neurology, Fondazione IRCCS Cà Granda Ospedale Maggiore Policlinico, Milan, Italy; 2 Department of Neuroradiology, Fondazione IRCCS Cà Granda Ospedale Maggiore Policlinico, Milan, Italy; 3 Department of Acute Brain and Cardiovascular Injury, Institute for Pharmacological Research Mario Negri IRCCS, Milan, Italy; 4 NEUROFARBA Department, Neuroscience Section, University of Florence, Florence, Italy; 5 Stroke Unit, Careggi University Hospital, Florence, Italy; 6 Department of Neurosurgery, IRCCS Istituto Giannina Gaslini, Genoa, Italy; 7 Unit of Genomics and Clinical Genetics, IRCCS Istituto Giannina Gaslini, Genoa, Italy; 8 Department of Neurosurgery, Fondazione Policlinico Universitario A. Gemelli IRCCS, Università Cattolica del Sacro Cuore, Rome, Italy; 9 Division of Neurosurgery, Department of Translational Biomedicine and Neurosciences (DiBraiN), University "Aldo Moro" of Bari, Bari, Italy; 10 Clinica Neurochirurgica, Dipartimento di Medicina, Chirurgia e Odontoiatria, Scuola Medica Salernitana, Università degli Studi di Salerno, Salerno, Italy; 11 Neuroradiology Unit, Department of Experimental and Clinical Biomedical Sciences, University of Florence, Florence, Italy; 12 Neuroradiology Unit, IRCCS Istituto Giannina Gaslini, Genoa, Italy; 13 Neurosurgery, Fondazione IRCCS Cà Granda Ospedale Maggiore Policlinico, Milan, Italy; 14 Department of Pathophysiology and Transplantation, University of Milan, Milan, Italy; 15 UOC Radiodagnostica Universitaria Policlinico di Bari, Bari, Italy

**Keywords:** cerebral cavernous malformation, familial cerebral cavernous malformation, intracerebral heamorrhage, focal neurological deficit, registry

## Abstract

**Background:**

Familial cerebral cavernous malformations (fCCMs) are a rare genetic autosomal dominant cerebrovascular disease characterized by multiple cerebral and spinal angiomas. The condition is caused by mutations in KRIT1 (CCM1), CCM2 (malcavernin), or PDCD10 (CCM3) and may lead to intracerebral hemorrhage (ICH) or non-hemorrhagic focal neurological deficits (FNDs), potentially leading to severe disability and even death. To date, little is known about disease progression, and tools to identify patients at higher risk are lacking.

**Methods:**

Pediatric and adult fCCM patients, whether symptomatic or asymptomatic, will be enrolled and followed annually over a 2-year period. Participants will undergo clinical assessments, blood sampling, and 3 T brain MRI scans at baseline, 12 months, and 24 months. The primary outcome is the new occurrence of symptomatic ICH or FNDs attributable to CCMs over 24 months. Patient characteristics will be assessed for the primary and secondary endpoints and illustrated using Kaplan–Meier curves and Cox proportional hazard regressions. This trial is registered with ClinicalTrials.gov, NCT06983132 and is currently recruiting participants.

**Discussion/conclusion:**

Despite increasing efforts in basic and clinical research and an improved understanding of the pathogenic mechanisms underlying fCCM, tools to predict disease progression, identify at-risk individuals, and pinpoint effective therapeutic targets are still lacking. This study aims to create the largest Italian cohort of fCCM patients, who will be monitored closely over time to collect data that may help identify risk factors and disease trajectories. The collection of standardized information on clinical and radiological evolution, along with results from circulating biomarkers, will help address the complexities of the disease and may suggest potential reliable markers of disease progression.

**Clinical trial registration:**

ClinicalTrials.gov, identifier NCT06983132.

## 1 Introduction

Familial cerebral cavernous malformations (fCCMs) are a rare genetic autosomal dominant cerebrovascular disease characterized by microvessel dilatations with deficient blood–brain barriers and disrupted inter-endothelial junctions, resulting in vessel hyperpermeability and fragility. CCMs may result in intracerebral hemorrhage (ICH) or non-hemorrhagic focal neurological deficits (FNDs) due to the growth of lesions in eloquent brain areas, potentially leading to severe disability and even death. Additionally, the disease may cause seizures and recurrent headaches. Familial CCMs (fCCMs) are a rare condition caused by loss-of-function mutations in one of three independent genes: KRIT1 (CCM1), malcavernin (CCM2), or programmed cell death 10 (PDCD10/CCM3). To date, no curative treatment exists, and little is known about clinical, radiological, or blood biomarkers that can monitor disease progression or identify patients at higher risk of serious clinical events.

Despite improved understanding of its pathophysiology, reliable biomarkers to monitor progression and stratify risk are still lacking. This underscores the need for longitudinal studies integrating clinical, imaging, and biomarker data.

Brain MRI is a valid tool to diagnose and monitor CCM lesions, but it is expensive and may be poorly tolerated by some patients. Circulating biomarkers could complement imaging as a tool for patient classification and monitoring disease progression.

Based on our group’s previous experience in the context of the Treat_CCM phase 2 clinical trial (1, 2) and a review of the literature, we identified a set of biochemical parameters that may predict CCM progression, offering insights into the underlying biological mechanisms and potential therapeutic approaches for this rare condition.

## 2 Methods and analysis

### 2.1 Study design

CCM_Italia is a multicenter, non-profit, prospective, observational, non-pharmacological study. Pediatric and adult patients with genetically confirmed fCCMs will be enrolled according to the inclusion and exclusion criteria designed to include a large representative cohort reflecting the heterogeneity of fCCM, ranging from asymptomatic to high-risk cases. CCM_Italia aims to identify clinical, imaging, and biochemical predictors of disease progression.

Patients will be enrolled during the clinical visits planned with their treating physician. As a non-interventional registry, participation will not influence routine clinical care or surgical decisions. Patients’ enrollment will take place after successful screening and confirmation of eligibility at Fondazione IRCCS Cà Granda Ospedale Maggiore Policlinico (Policlinico Milano) in Milan, Azienda Ospedaliero-Universitaria Policlinico in Bari, Azienda Ospedaliero Universitaria San Giovanni e Ruggi d’Aragona in Salerno, and Azienda Ospedaliero Universitaria Careggi in Firenze. Pediatric patients’ enrollment will take place primarily at IRCCS Pediatrico Istituto Giannina Gaslini in Genova.

The study will combine longitudinal clinical data with MRI as well as peripheral blood collection at baseline (T0) and after 1 (T1) and 2 (T2) years to identify circulating biomarkers for assessing clinical evolution and the incidence of adverse CCM-related clinical events in these patients. MRI will be performed according to a standardized protocol, and the obtained images will be assessed centrally by expert radiologists at Fondazione IRCCS Cà Granda Ospedale Maggiore Policlinico. Biological samples will be stored long-term in the UN EN ISO 9001:2015-certified “SATURNE” biobank at Istituto di Ricerche Farmacologiche Mario Negri IRCCS.

#### 2.1.1 Inclusion criteria

Patients who simultaneously meet the following criteria will be included:

1. Adult and pediatric patients with familial cerebral cavernous malformations documented by multiple CCM lesions on MRI and a confirmed pathogenic mutation in CCM1, CCM2, or CCM3;
2. Symptomatic or asymptomatic;
3. Life expectancy at least as long as the study follow-up duration;
4. Written informed consent provided by the patient (or caregivers in the case of minors) to participate in the study.

#### 2.1.2 Exclusion criteria

The patient who does not meet the inclusion criteria mentioned above or meet any of the following conditions will not be eligible to participate in the study:

1. Implanted pacemaker or any other condition preventing MRI;
2. Inability to cooperate with the study procedures.

#### 2.1.3 Exit study criteria

A patient will be considered discontinued from the study only if they withdraw consent for follow-up at the participating center or are lost to follow-up after all means of contact have been exhausted. Surgical correction of angiomas during follow-up will not necessitate discontinuation from the study. In cases of discontinuation, the status of the patient at the last visit or last available contact will be used for the final analysis. Vital status may be ascertained through public records if all other means of contact fail.

### 2.2 Study procedures

#### 2.2.1 Patient enrollment and data collection procedures

Patients with successful screening (presence of inclusion criteria and absence of exclusion criteria) will be asked to sign informed consent. Each participant, at the time of enrollment, will be assigned a unique code so that only local investigators can trace the identity of enrolled subjects.

All patients who are included in the CCM_Italia cohort will undergo an annual clinical assessment according to clinical practice (T0-T1-T2). This assessment will be performed in the outpatient clinic at the referral hospital. During these routine follow-up patient visits, information will be collected on the following events: ICH, FNDs, seizures, recurrent headaches, hospitalizations, and death.

In accordance with clinical practice, an annual brain MRI will be performed in the Neuroradiology Unit of the referral hospital, preferably on the same day as the clinical assessment. If this is not possible, the MRI will be scheduled within 1 month for the inclusion visit T0 and within ± 40 days for follow-up visits (T1-T2). The MRI protocol will include sagittal 3D T1-weighted turbo field echo, sagittal 3D T2-weighted turbo spin echo, sagittal 3D fluid-attenuated inversion recovery, axial diffusion-weighted imaging, axial susceptibility-weighted imaging, axial T2-weighted gradient echo, and optional diffusion tensor imaging and axial spoiled 3D multi-echo gradient-echo sequences (SGRE) for quantitative susceptibility mapping (QSM). The current protocol is in line with standard clinical practice. The collected MRIs will be centrally assessed at the MRI core laboratory of IRCCS Fondazione Ca′ Granda Ospedale Maggiore Policlinico by blinded personnel. The following MRI variables will be recorded at baseline and follow-up examinations: CCM lesion characteristics including number, signal (8), size, and location of CCMs and size modifications, signs of *de novo* bleeding, and number of *de novo* lesions at 12 and 24 months. Image examples of each Zabramski type of lesion will be provided to ensure that raters understand the Zabramski classification system. A standardized interpretation of MR imaging features on T1, T2, and SWI images that are relevant to classification will be discussed. Raters will independently classify a set of 15 MRI cases and then review any discrepancies. Interrater reliability will be assessed using a separate set of 30 MRI cases, which raters will classify independently while remaining blinded to each other’s assessments. Cohen’s kappa will be calculated.

Advanced MRI techniques such as diffusion tensor imaging (DTI) and QSM, which are not routinely performed in the follow-up of CCM patients, will be applied if available at the center and analyzed at IRCCS Fondazione Ca′ Granda Ospedale Maggiore Policlinico in the MRI core laboratory to assess iron deposition (3) and structural integrity. DTI and QSM do not require contrast media injection. This analysis does not impose any extra burden on patients, as they already undergo an annual MRI as part of clinical practice.

In addition to the standard annual clinical assessment described above, adult patients will be asked to complete specific questionnaires for patient-reported outcome measures (PROMs) and patient-reported experience measures (PREMs) on an annual basis. These include the following standardized questionnaires: the Beck Depression Inventory-II (BDI-II) for depression; the State–Trait Anxiety and Quality of Life for anxiety; and Short Form 36 (SF-36) for quality of life, divided into the physical and mental component scales (PCS and MCS). Furthermore, cognitive function and disability will be assessed using specific questions in the questionnaire, which will be completed by the patient during the clinical visit; this usually takes 10–15 min (4). For minors, these questionnaires will not be applicable unless they have been specifically validated for their age category.

All patients’ clinical and radiological data, as well as outcomes of questionnaires and specific information on clinical events, will be de-identified and recorded in electronic case report files on the REDCap platform.

#### 2.2.2 Biological sample collection and analysis

In addition to the standard annual clinical assessment, patients will undergo blood sample collection at the same time as their annual clinical visit. Three vials, containing a total of 15 mL of blood, will be collected at the outpatient clinic of the referral hospital during clinical visits at baseline (T0), 12 months (T1), and 24 months (T2) to obtain whole blood, EDTA plasma, and citrate plasma samples. Blood samples will be drawn from a peripheral vein into vacuum tubes containing sodium citrate and EDTA-K3 anticoagulants. The tubes will then be gently inverted 5–6 times and placed upright at room temperature. One aliquot of whole blood (1 mL) will be transferred into a cryovial. After collection, blood samples will be centrifuged within 2 h at 4 °C for 15 min at 2,000 × *g*. Plasma will then be aliquoted and stored locally at −70 °C before being periodically shipped on dry ice with a dedicated courier to the certified ISO 9001:2015 SATURNE biobank at the Mario Negri Institute for Pharmacological Research in Milan, Italy, where they will be centralized and stored long-term in a − 70 °C freezer under controlled conditions for further analysis. Standard operating procedures for sample collection, processing, and storage, as well as the necessary materials for blood collection, will be shared with all clinical centers participating in the study. All samples will be de-identified with a unique 6-digit code. All assays will be conducted according to the manufacturer’s instructions.

### 2.3 Measurements and outcomes

#### 2.3.1 Primary objectives

- To evaluate the occurrence of new CCM-related events, which are defined as intracerebral hemorrhage (ICH) or focal neurological deficits (FNDs). FNDs will be assessed by treating clinicians (neurologist or neurosurgeon) based on clinical and neuroradiological evaluation (cerebral CT or cerebral MRI).

#### 2.3.2 Secondary objectives

- To evaluate the new occurrence of other CCM-related clinical events, including seizures and recurrent headaches. Seizures, an important aspect of fCCMs, are assessed as a secondary endpoint rather than a primary endpoint, as their onset may be influenced by anti-convulsant therapy.
- To identify MRI biomarkers of disease severity and disease progression, including the number of CCM lesions, size of CCMs, signal of CCM lesions according to the Zabramski classification, lesion location, size modifications, signs of new bleeding, and the number of *de novo* CCM lesions at 12 and 24 months.
- To identify circulating biomarkers related to disease activity in fCCM subjects.
- To evaluate global cognitive function, disability, and health-related quality of life of fCCM patients.

#### 2.3.3 Event adjudication

A Clinical Event Committee will be established to assess all outcome events. Once an endpoint event (ICH or FND) is reported, the Study Secretariat will collect the clinical documentation and share it with independent experts to centrally verify the event. The independent Clinical Event Committee will oversee the adjudication of all primary events in accordance with current guidelines (5).

#### 2.3.4 Data management

Each participant will be assigned a unique code at the time of enrollment. Data de-identification will occur in such a way that individuals accessing the database will not be able to trace back to the identities of the subjects in any way. Only local researchers will be able to trace back to the identities of the enrolled subjects.

The data required for the study will be recorded in a dedicated eCRF within a Data Management System validated according to national regulations, provided by Institute for Pharmacological Research Mario Negri IRCCS. The platform used will be Research Electronic Data Capture (REDCap). In this study, REDCap will provide:

1. user-level identification with specific restrictions based on study roles,
2. real-time data validation and integrity control,
3. patient de-identification prior to data export, and
4. centralized data storage with daily backups on a secure server within the IRFMN infrastructure.

#### 2.3.5 Sample size calculation

The primary endpoint, the new occurrence of CCM-related clinical events, defined as ICH and FNDs, excluding seizures, is expected in approximately 5.6% of the population based on the Treat_CCM trial (1). Assuming that 5.6% of subjects in the CCM_Italia cohort will develop a CCM-related clinical event, at least 100 patients (including a dropout of 8%) are needed to estimate this proportion with 95% confidence and 4.7% absolute precision.

According to the Treat_CCM study, 55% of fCCM patients are expected to experience increased disease activity. Dividing the population by the median level of a biomarker or imaging marker of interest (resulting in 2 groups of 50 individuals) would provide us a statistically significant result if the HR of this biomarker is at least 2.15 (power 80%, *α* = 0.05) (6, 7).

#### 2.3.6 Statistical analysis

Baseline characteristics will be presented for the total population and by relevant variables (e.g., sex, age categories, and number of CCM lesions). Relevant patient subgroups, such as gene mutation, age groups, and presence of baseline symptoms, will be compared by descriptive univariate statistics. Prior experience of the consortium has shown that data are quite complete, and imputation for missing values was not performed.

##### 2.3.6.1 Primary endpoint

Patient characteristics will be compared using descriptive analyses to compare patients who reach the primary endpoint—the new occurrence of CCM-related clinical events, defined as ICH and FNDs, excluding seizures—to those who do not. The Kaplan–Meier curves will be constructed to assess the onset of the primary endpoint over time.

##### 2.3.6.2 Secondary endpoint

*Clinical endpoints*: individual components of the primary endpoint, the incidence of either ICH or FNDs, and other clinical secondary endpoints such as seizures, headache, or any hospitalization, will be assessed by means of descriptive analyses. In addition, the Kaplan–Meier curves will be constructed to illustrate the occurrence of clinical events over time.

*Disease activity*: In order to explore the discriminatory power of biomarkers, we will assess a secondary endpoint: disease activity. This will be defined as CCM-related adverse events or the development of at least 5 new CCM lesions during the 2 years of observation. Based on (2), we expect at least 55% of the participating fCCM patients to be classified as “active.” Patients will be divided by the median level of a biomarker or imaging marker of interest. Logistic regression analyses will be performed to identify circulating biomarkers independently associated with increased disease activity. All outcomes will be reported with 95% confidence intervals, and in the case of -omics assessments, corrections for multiple testing will be applied.

*Questionnaires*: Global cognitive function, depression, anxiety, and health-related quality of life will be assessed in adult patients at multiple time points, and any changes over time will be described and assessed using repeated measures ANOVA.

*Neuroimaging*: Different vascular lesion characteristics, as measured by MR imaging, will be assessed for the total population and by relevant characteristics:

- CCM lesions (number).
- CCM new lesions (number).
- Location of lesions (number of CCM lesions in each of the following sites: the cerebellum, the brainstem, the basal ganglia and thalami, the right cerebral hemisphere, and the left cerebral hemisphere).
- Radiological characteristics (size and number of CCM lesions) classified according to Zabramski classification: type I, II, III, and IV (7). The evolution of MRI markers, such as the number of new lesions, the size modification of lesions, and the signs of *de novo* bleeding, will be described. Changes over time will be assessed by means of generalized linear models with a Poisson distribution to account for the count nature of the data. Corrections for multiple testing will be applied to account for type 1 errors across multiple MRI outcomes. The assessment of changes over time will be performed for all patients included in the cohort as a change from baseline to 2-year follow-up.

## 3 Discussion

In this study, a large number of adult and pediatric subjects affected by fCCMs will be observed for 24 months. Despite efforts in basic and clinical research and an improved understanding of the pathogenic mechanisms underlying the disease, predicting disease progression and identifying at-risk individuals and effective therapeutic targets remains challenging. This is particularly true for complex diseases, where genetic and environmental factors interact, and for rare conditions.

To tackle these challenges, this study will collect data that may help identify risk factors and disease trajectories. Moreover, we will collect information searching for specific biomarkers and imaging markers that could help identify at-risk patients and monitor disease progression and will combine expertise from various disciplines (biology, genetics, epidemiology, neurology, neurosurgery, and neuroradiology) to address the complexities of the disease.

The identification of biomarkers for disease progression is a crucial aspect in the management of multiple cerebral cavernous malformations (CCMs) and could also have significant implications for the treatment of sporadic malformations in terms of:

- Monitoring disease progression: Reliable biomarkers can provide objective indicators of the disease status and its progression over time. This is particularly important for CCMs, which have a variable course. Improved quantification of disease progression may optimize the timing and methods of intervention and establish more effective follow-up protocols.
- Discovery of novel therapeutic targets: Biomarkers may reveal the pathogenic mechanisms underlying CCMs. A better understanding of the biological processes involved in the formation and progression of cavernous malformations may lead to the identification of new therapeutic targets.
- Establishment of personalized treatment: Identification of specific biomarkers and novel therapeutic targets may help the development of personalized treatment strategies. Biomarkers that indicate disease severity or the likelihood of hemorrhage can enable clinicians to tailor strategies based on individual patient characteristics. This approach may lead to a better selection of patients undergoing surgery and of those requiring closer monitoring, thereby improving clinical outcomes.
- Reduction of adverse events: In CCM patients, recurrent hemorrhages can result in significant disabilities and even death. Identifying biomarkers that indicate a high risk of complications, such as hemorrhage, enables the implementation of more effective preventive measures.
- Biomarker research can stimulate further clinical and preclinical studies, inform the design of innovative clinical trials, and create a virtuous cycle of scientific discovery and therapeutic improvement.

The significance of our project lies primarily in the dataset generated, which will represent the largest Italian prospective registry on the natural history of fCCMs assessed through combined approaches (e.g., clinical, MRI, and biochemical). Moreover, building on the biomarkers identified in the Treat_CCM clinical trial (#NCT03589014), this project will expand and validate the understanding of fCCMs.

Beyond the scope of the project, this dataset, which will continue to expand, will generate new insights into CCMs, improving clinicians’ expertise and patients’ treatment. The creation of a large standardized database will facilitate data access and promote collaborations among health professionals in case of future therapeutic trials. Including not only adults but also children with fCCMs in this observational cohort will expand knowledge about disease progression.

Moreover, the identification of biomarkers for disease progression is a fundamental step in guiding the clinical management of this challenging condition and enhancing clinical outcomes and quality of life. These biomarkers may also inform the treatment of sporadic malformations, a far more common condition.

## Group member of CCM_Italia collaborators

Fondazione IRCCS Cà Granda Ospedale Maggiore Policlinico: Marco Locatelli, Silvia Lanfranconi, Elisa Scola, Luigi Remore, Giorgio Conte, Giulio A. Bertani, Maria Chiara Palmieri, Alessandra Previtali, Roberto Del Bo, Stefania P. Corti, Giacomo P. Comi, Dario Ronchi, and Francesca Lazzaroni.

Istituto di Ricerche Farmacologiche Mario Negri: Deborah Novelli, Elisa R. Zanier, Jennifer M. T. A. Meessen, Antonella Vasamì, Enrico Nicolis, Giorgia Contino, Roberto Bernasconi, and Roberto Latini.

Policlinico Bari: Walter Marani, Francesco Signorelli, Maria Teresa Bozzi, and Maria Elena Solarino.

Salerno: Giorgio Iaconetta, Giovanni Torelli, and Rita Caliulo.

Careggi University Hospital: Anna Poggesi, Francesca Pescini, Filippo Fratini, Enrico Fainardi, Stefano Chiti, Andrea Ginestroni, and Enrico Fainardi.

IRCCS Istituto Giannina Gaslini: Marco Pavanello, Valeria Capra, Mariasavina Severino, and Ferruccio Romano.

Roma: Quintino Giorgio D’Alessandris, Pier Paolo Mattogno, and Carmelo L. Sturiale.
